# Gentisic acid attenuates pressure overload‐induced cardiac hypertrophy and fibrosis in mice through inhibition of the ERK1/2 pathway

**DOI:** 10.1111/jcmm.13869

**Published:** 2018-09-06

**Authors:** Simei Sun, Hae Jin Kee, Li Jin, Yuhee Ryu, Sin Young Choi, Gwi Ran Kim, Myung Ho Jeong

**Affiliations:** ^1^ Heart Research Center Chonnam National University Hospital Gwangju Korea; ^2^ Hypertension Heart Failure Research Center Chonnam National University Hospital Gwangju Korea; ^3^ Molecular Medicine Brain Korea 21 PLUS Chonnam National University Graduate School Gwangju Korea; ^4^ The Second Affiliated Hospital & Yuying Children's Hospital Wenzhou Medical University Wenzhou China

**Keywords:** cardiac fibrosis, cardiac hypertrophy, ERK1/2 MAPK signalling pathway, GATA4/Sp1, gentisic acid, transverse aortic constriction

## Abstract

We previously reported that gentisic acid (2,5‐dihydroxybenzoic acid) is the third most abundant phenolic component of *Dendropanax morbifera* branch extracts. Here, we investigated its effects on cardiac hypertrophy and fibrosis in a mouse model of pressure overload and compared them to those of the beta blocker bisoprolol and calcium channel blocker diltiazem. Cardiac hypertrophy was induced in mice by transverse aortic constriction (TAC). Beginning 2 weeks after this procedure, the mice were given daily intraperitoneal injections of gentisic acid (100 mg/kg/d), bisoprolol (5 mg/kg/d) or diltiazem (10 mg/kg/d) for 3 weeks. Cardiac hypertrophy was evaluated by the heart weight‐to‐body weight ratio, the cardiomyocyte cross‐sectional area after haematoxylin and eosin staining, and echocardiography. Markers of cardiac hypertrophy and fibrosis were tested by reverse transcription‐quantitative real‐time polymerase chain reaction, western blotting and Masson's trichrome staining. The suppressive effects of gentisic acid treatment on TAC‐induced cardiac hypertrophy and fibrosis were comparable to those of bisoprolol administration. Cardiac hypertrophy was reversed and left ventricular septum and posterior wall thickness were restored by gentisic acid, bisoprolol and diltiazem treatment. Cardiac hypertrophic marker gene expression and atrial and brain natriuretic peptide levels were decreased by gentisic acid and bisoprolol, as were cardiac (interstitial and perivascular) fibrosis and fibrosis‐related gene expression. Cardiac hypertrophy‐associated upregulation of the transcription factors GATA4 and Sp1 and activation of extracellular signal‐regulated kinase 1/2 were also negated by these drugs. These results suggest that gentisic acid could serve as a therapeutic agent for cardiac hypertrophy and fibrosis.

## 1 INTRODUCTION

Cardiac hypertrophy and fibrosis occur as adaptive or compensatory responses to overcome increased cardiac wall stress induced by a variety of stimuli, including hypertension, myocardial infarction, and valvular heart disease. However, sustained stimulation results in a pathological state, such as heart failure. Cardiac hypertrophy is regarded as a critical cardiovascular risk factor and is typically associated with certain morphological changes. Preventing its initial development or escalation into heart failure is a key therapeutic strategy. Transverse aortic constriction (TAC) in mice or rats is the most commonly used experimental method of modelling cardiac hypertrophy and fibrosis in animals.^1^ Currently employed treatments for patients with cardiac hypertrophy include calcium channel blockers, beta blockers, angiotensin‐converting enzyme inhibitors, angiotensin II receptor blockers, and diuretics.^2^, ^3^

The mitogen‐activated protein kinase (MAPK) signalling cascade is associated with pressure overload‐induced cardiac hypertrophy. Extracellular signal‐regulated kinase (ERK), c‐Jun N‐terminal kinase (JNK), and p38 MAPK are simultaneously activated after TAC surgery,^4^ leading to cardiac hypertrophy, and overexpression of the activated form of mitogen‐activated protein kinase kinase 1 (MKK1), a kinase upstream of ERK1, has also been shown to result in this condition.^5^ Furthermore, the importance of ERK to cardiomyocyte hypertrophy has been supported by the results of another study in which the MKK1/2 inhibitor U0126 was employed.^6^ In contrast to ERK, the role of JNK in cardiac hypertrophy is ambiguous.^7^ For example, cardiac hypertrophy occurs because of MKK7 overexpression^8^; however, the opposite phenomenon is caused by MKK4.^9^ Moreover, p38 MAPK induces cardiac hypertrophy in cardiomyocytes infected with activated MKK3 beta or MKK6 beta.^10^, ^11^ Overall, MAPK signalling is associated with cardiac hypertrophy. However, it may vary depending on the experimental conditions. Therefore, we need to investigate MAPK signalling in our TAC experimental conditions.

Gentisic acid (2,5‐dihydroxybenzoic acid) is a bioactive polyphenol found in many food plants (ripe bitter melon, gooseberries, kiwifruit, chicory and buckwheat, among others),^12^ as well as *Dendropanax morbifera* branch extracts.^13^ Gentisic acid is a metabolite of aspirin (acetylsalicylic acid)^14^ and demonstrates a variety of biological properties, including the inhibition of low‐density lipoprotein oxidation,^15^ and protection against gamma radiation exposure^16^ and cyclophosphamide‐induced genotoxicity and hepatotoxicity.^17^ It has also been shown to have no cytotoxic or antiproliferative effects on hepatic tumour cells.^18^ A new class of fibroblast growth factor inhibitors potentially relevant to the treatment of cancer and angiogenesis‐related diseases has been defined based on gentisic acid.^19^ In addition, hydroxybenzoic acids have been reported to attenuate factors associated with cardiovascular diseases, such as hypertension, atherosclerosis and dyslipidaemia.^20^ Recently, we demonstrated that gentisic acid is the third most abundant polyphenol compound in *Dendropanax morbifera* branch extracts and reduces isoproterenol‐induced cardiomyocyte hypertrophy (paper under review). However, its influence on cardiac hypertrophy and fibrosis in vivo has not yet been reported.

We performed this study to investigate whether gentisic acid can ameliorate cardiac hypertrophy and fibrosis induced by pressure overload in mice. Our results showed that gentisic acid treatment did indeed attenuate cardiac hypertrophy and fibrosis, through down‐regulation of Sp1/GATA4 and MAPK signalling. The therapeutic effect of gentisic acid was comparable to that of treatment with bisoprolol.

## 2 MATERIALS AND METHODS

### 2.1 TAC and experimental groups

All animal procedures were approved by the Animal Experimental Committee of Chonnam National University Medical School (CNU IACUC‐H‐2018‐4) and were carried out according to the Guide for the Care and Use of Laboratory Animals (US National Institutes of Health Publications, 8th edition, 2011). Male CD‐1 mice (6 weeks old and with an average weight of 30 g) were anaesthetized with an intraperitoneal injection of ketamine (120 mg/kg) and xylazine (6.2 mg/kg), before undergoing either a sham operation or TAC. TAC was performed as follows. The mice were intubated with an endotracheal tube connected to a rodent ventilator. After exposure of the aortic arch, the thymus was removed. The transverse aortic arch was then ligated between the brachiocephalic and left common carotid arteries with a 7‐0 silk suture and a placing 27 G needle. Mice in the sham surgery group underwent the same operation, with the omission of aorta ligation. The success of the TAC procedure was then confirmed by echocardiography. The mice were randomly divided into the five following groups: sham + DMSO vehicle (n = 10), TAC + DMSO vehicle (n = 14), TAC + gentisic acid (n = 13; 100 mg/kg/d), TAC + bisoprolol (n = 8; 5 mg/kg/d) and TAC + diltiazem (n = 7; 5 mg/kg/d). TAC surgery and subsequent experimental analyses for all the groups were performed in a blinded manner. Gentisic acid, bisoprolol and diltiazem were dissolved in DMSO. After drug administration for 3 weeks, the mice were euthanized.

### 2.2 Echocardiography

Echocardiography was performed using a Vivid S5 echocardiography system (GE Healthcare, Chicago, IL, USA) with a 13‐MHz linear array transducer. Mice were anaesthetized with an intraperitoneal injection of tribromoethanol (Avertin; 114 mg/kg) before the procedure. M‐mode (2‐D guided) images and recordings were acquired from the long‐axis view of the left ventricle at the level of the papillary muscles. The thickness of the anterior and posterior wall was measured from the images, whereas the left ventricular end‐diastolic diameter (LVEDD) and left ventricular end‐systolic diameter (LVESD) were measured from the M‐mode recordings. Fractional shortening (FS) was calculated as follows: FS (%) = (LVEDD − LVESD) × 100/LVEDD.

### 2.3 Histology and Masson's trichrome staining

The hearts of mice in the sham, TAC and TAC + drugs groups were fixed with 4% paraformaldehyde and embedded in paraffin. The paraffin‐embedded tissues were then cut into 3‐μm sections, deparaffinized with xylene and rehydrated with different grades of ethanol. To measure cardiomyocyte area, tissue sections were stained with haematoxylin and eosin (H&E).

To measure cardiac fibrosis, we performed Masson's trichrome staining as described previously, with some modifications.^21^ Briefly, heart sections were incubated with prewarmed Bouin's solution for 30 minutes to intensify the final coloration. Nuclei were stained by exposure to Weigert's haematoxylin for 20 minutes, and cytoplasm and muscle were stained by incubation with Biebrich scarlet‐acid fuchsin solution for 20 minutes. The tissue sections were treated with phosphomolybdic‐phosphotungstic acid solution for 10 minutes, followed by aniline blue solution for 10 minutes. After incubation with 1% acetic acid for 30 seconds, the sections were dehydrated with ethanol and xylene. Digital images were obtained with a microscope at a magnification of 400×. Interstitial and perivascular fibrosis were quantified by calculating the percentage area of collagen staining using a Nikon Eclipse 80*i* microscope and NIS Elements software (Nikon Corp., Tokyo, Japan).

### 2.4 Reagents

Gentisic acid (2,5‐dihydroxybenzoic acid; catalogue number 149357) was purchased from Sigma (Billerica, MA, USA).^22^ Bisoprolol fumarate (S1206) and diltiazem HCl (S1865) were purchased from Selleck Chemicals (Houston, TX, USA).^23^, ^24^, ^25^

### 2.5 Reverse transcription‐quantitative real‐time polymerase chain reaction (RT‐qPCR)

Total RNA was isolated from heart tissue with TRIzol reagent (Invitrogen/Life Technologies, Carlsbad, CA, USA), and 1 μg was reverse transcribed with TOPscript RT DryMIX (Enzynomics, Daejeon, South Korea). mRNA levels were then quantified using a SYBR Green PCR kit (Enzynomics). The PCR primers used in this study are given in Table 1.

**Table 1:** Echocardiographic parameters in mouse hearts

	Sham (n = 10)	TAC (n = 14)	TAC+gentisic acid (n = 13)	TAC+bisoprolol (n = 8)	TAC+diltiazem (n = 7)
At basal level					
LVSd (mm)	0.65 ± 0.05	0.65 ± 0.05^NS^	0.67 ± 0.05	0.67 ± 0.05	0.67 ± 0.05
LVPWd (mm)	0.65 ± 0.05	0.65 ± 0.05^NS^	0.62 ± 0.04	0.62 ± 0.04	0.65 ± 0.05
LVESD (mm)	2.02 ± 0.24	1.95 ± 0.21^NS^	2.00 ± 0.26	2.00 ± 0.12	1.97 ± 0.07
LVEDD(mm)	3.58 ± 0.24	3.68 ± 0.13^NS^	3.78 ± 0.22	3.73 ± 0.24	3.77 ± 0.18
FS (%)	45.93 ± 5.11	47.56 ± 6.56^NS^	49.59 ± 6.50	48.86 ± 5.00	45.89 ± 2.88
5 weeks post‐TAC					
LVSd (mm)	0.70 ± 0.00	0.99 ± 0.06***	0.75 ± 0.05###	0.74 ± 0.05###	0.74 ± 0.05##
LVPWd (mm)	0.70 ± 0.00	0.99 ± 0.05***	0.75 ± 0.07###	0.75 ± 0.05##	0.74 ± 0.08##
LVESD (mm)	2.12 ± 0.22	2.41 ± 0.51^NS^	2.12 ± 0.32	2.41 ± 0.35	2.44 ± 0.24
LVEDD (mm)	3.64 ± 0.31	4.03 ± 0.47^NS^	3.78 ± 0.27	4.06 ± 0.37	4.16 ± 0.32
FS (%)	41.65 ± 3.81	39.85 ± 6.87^NS^	43.76 ± 6.02	41.67 ± 4.37	41.84 ± 3.00

Values are mean ± SE.

TAC, transverse aortic constriction; LVPWd, left ventricular posterior thickness; LVESD, left ventricular end‐systolic diameter; LVEDD, left ventricular end‐diastolic diameter; FS, fractional shortening; LVSd, left ventricular septum thickness.

***P < 0.001 compared with sham, ^#^^#^ *P* < 0.01 and ^#^^#^^#^ *P* < 0.001 compared with TAC, NS (not significant) compared with sham.

### 2.6 Western blotting

Total protein was extracted from heart tissues using RIPA lysis buffer (150 mmol/L NaCl, 1% Triton X‐100, 1% sodium deoxycholate, 50 mmol/L Tris‐HCl at pH 7.5, 2 mmol/L EDTA, 1 mmol/L PMSF, 1 mmol/L DTT, 1 mmol/L Na_3_VO_4_ and 5 mmol/L NaF) containing a protease inhibitor cocktail (Calbiochem/EMD Millipore, Billerica, MA, USA). Proteins were subjected to SDS‐PAGE and transferred to polyvinylidene difluoride membranes, which were then blocked with 5% skim milk in TBST buffer (20 mmol/L Tris, 200 mmol/L NaCl and 0.04% Tween‐20) for 1 h at 25°C. The membranes were incubated overnight at 4°C with primary antibodies against collagen type III, fibronectin, connective tissue growth factor (CTGF), atrial natriuretic peptide (ANP), GATA4, Sp1, and GAPDH, before being exposed to anti‐rabbit or anti‐mouse horseradish peroxidase‐conjugated secondary antibodies (diluted 1:5000) for 1 h at 25°C. Protein bands were visualized using Immobilon western blotting detection reagents (EMD Millipore). Bio‐ID software (Vilber Lourmat, Eberhardzell, Germany) was used to quantify protein expression.

### 2.7 Statistical analysis

All data are expressed as means ± SE. Differences between groups were analysed by Kruskal‐Wallis test with Dunn's multiple comparison post hoc test using GraphPad Prism version 5 (GraphPad Software, La Jolla, CA, USA). *P* values <0.05 were considered statistically significant.

## 3 RESULTS

### 3.1 Gentisic acid inhibits cardiac hypertrophy in mice with TAC

**Figure 1:**
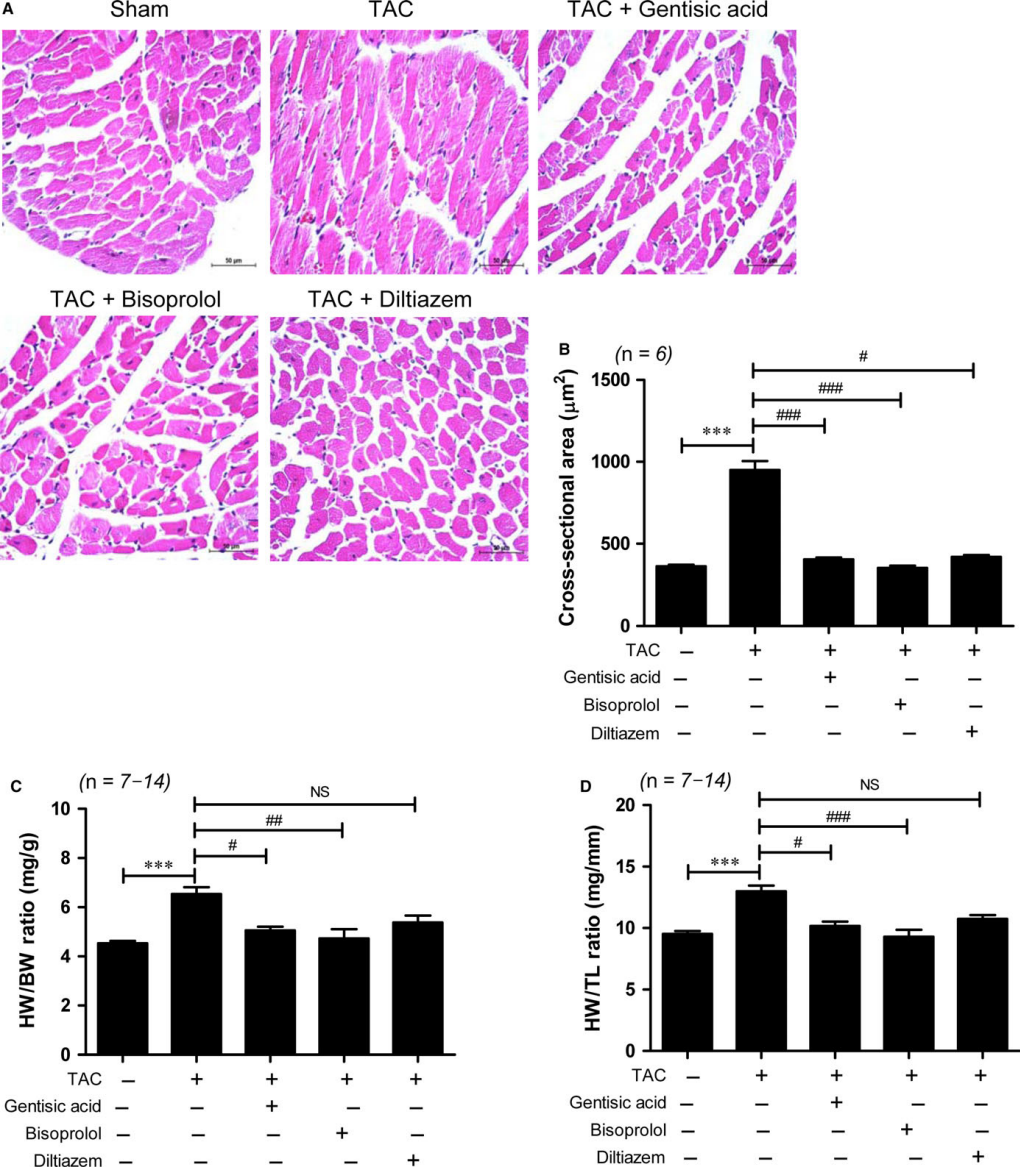
Gentisic acid reverses cardiac hypertrophy in mice with TAC. Cardiac hypertrophy developed in mice over the 2 weeks following the TAC operation, after which, they were administered vehicle (n = 14), gentisic acid (100 mg/kg/d; n = 13), bisoprolol (5 mg/kg/d; n = 8), or diltiazem (10 mg/kg/d; n = 7) for 3 weeks. A, Representative images of H&E staining of cardiac tissue from the sham, TAC, TAC + gentisic acid, TAC + bisoprolol, and TAC + diltiazem groups. Scale bar = 50 μm. B, Quantified cross‐sectional areas of cardiomyocytes from the sham and gentisic acid, bisoprolol, or diltiazem‐treated TAC groups. C and D, HW/BW and HW/TL ratios in each group (n = 7–14 per group). Data are means ± SE. ***P <* *0.001; ^#^ *P *<* *0.05, ^##^ *P *<* *0.01, and ^###^ *P *<* *0.001 vs the TAC group. TAC, transverse aortic constriction; HW/BW, heart weight to body weight; TL, tibia length; NS, not significant

To establish whether gentisic acid can ameliorate cardiac hypertrophy, mice were administered 100 mg/kg/d for 3 weeks beginning 2 weeks after TAC surgery. To compare the anti‐hypertrophic efficacy of gentisic acid to that of other drugs, we administered the calcium channel blocker diltiazem (10 mg/kg/d) and beta blocker bisoprolol (5 mg/kg/d) to other groups of mice for 3 weeks from the same time‐point. The doses used were chosen according to previously published studies.^26^, ^27^ H&E staining was performed to measure cardiomyocyte size. As shown in Figure 1A,B, all three drugs treatment in TAC mice resulted in a significant reduction in the cardiomyocyte cross‐sectional area. Furthermore, gentisic acid and bisoprolol treatments significantly suppressed cardiac hypertrophy, as determined by heart weight‐to‐body weight (HW/BW) and heart weight to tibia length (HW/TL) ratios (Figure 1C,D). Although the diltiazem was not significantly different in both HW/BW and HW/TL for TAC, it tended to decrease.

### 3.2 Gentisic acid blocks left ventricular hypertrophy in mice with TAC

**Figure 2:**
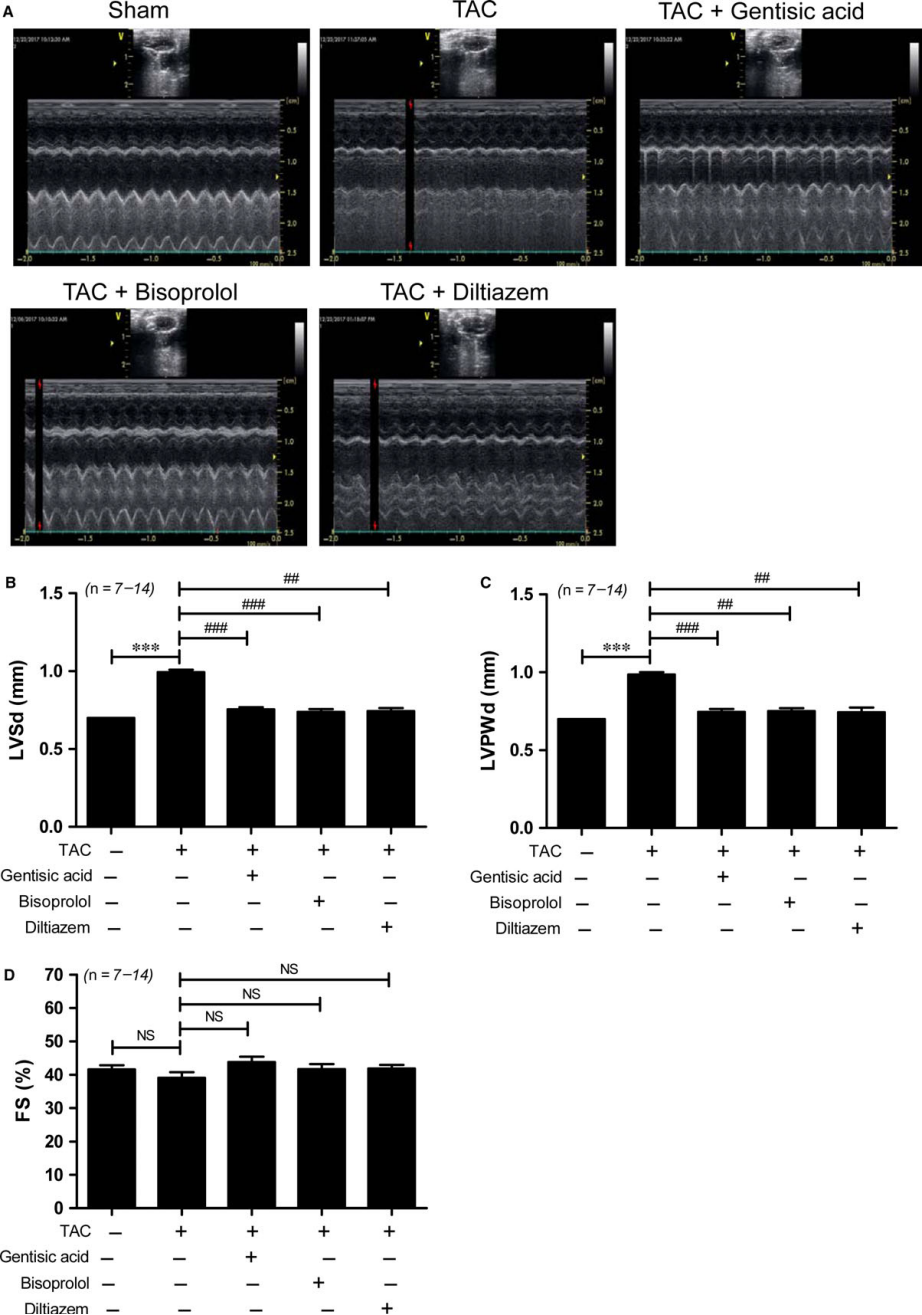
Gentisic acid attenuates left ventricular hypertrophy in mice with transverse aortic constriction (TAC). Left ventricular hypertrophy was determined by echocardiography after 3 weeks of drug administration beginning 2 weeks after the TAC procedure. A, Representative B‐mode and M‐mode echocardiograms are shown. B, Interventricular septum thickness (IVSd); C, left ventricular posterior thickness (LVPWd); and D, fractional shortening (FS) (%)

**Figure 3:**
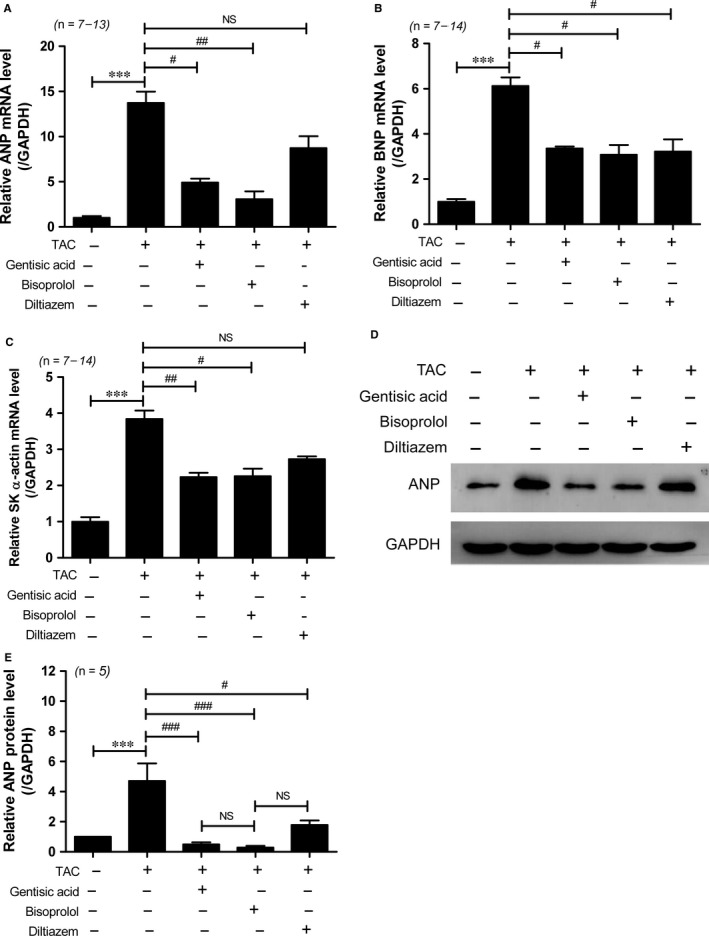
Gentisic acid reduces expression of hypertrophic marker genes in mice with transverse aortic constriction (TAC). Total RNA was isolated from the hearts of mice in the sham, TAC, TAC + gentisic acid, TAC + bisoprolol, and TAC + diltiazem groups. Levels of *Anp* (A), *Bnp* (B), and skeletal α‐actin (C) transcripts were determined by RT‐qPCR and normalized to those of *Gapdh*. Data are means ± SE. ***P <* *0.001; ^#^ *P *<* *0.05 and ^##^ *P *<* *0.01 vs the TAC group; NS, not significant. D, Representative western blot images showing atrial natriuretic peptide (ANP) protein levels in the hearts of mice. GAPDH was used as a loading control. The images were cropped from the same blot. E, ANP protein expression was quantified using densitometry. ***P <* *0.001; ^#^ *P *<* *0.05 and ^###^ *P *<* *0.001 vs the TAC group

Echocardiography was used to verify that TAC induced left ventricular hypertrophy. There were no changes in parameters between the groups before TAC surgery (Table 1). As shown in Figure 2A‐C, the thickness of the interventricular septum and left ventricular posterior wall were significantly increased in mice in the TAC group compared to those in the sham group (Table 1). As LVESD and LVEDD were no higher in the TAC groups than the sham group (Figure S1A,B), fractional shortening was not significantly decreased (Figure 2D). We next compared the effect of gentisic acid to that of bisoprolol and diltiazem on cardiac hypertrophic marker gene expression during TAC. *Anp*, brain natriuretic peptide (*Bnp*) and skeletal α‐actin mRNA levels were increased in mice subjected to TAC compared to those having undergone sham surgery. Treatment with gentisic acid and bisoprolol effectively attenuated the TAC‐induced cardiac expression of these hypertrophic markers (Figure 3A‐C). However, diltiazem did not significantly reduce *Anp* and skeletal α–actin mRNA expression. Similar trends in ANP protein expression were noted. Treatment with gentisic acid or bisoprolol effectively restricted the expression of ANP protein in hearts caused by TAC, as did administration of diltiazem (Figure 3D,E).

### 3.3 Gentisic acid reduces expression of the transcription factor genes *Sp1* and *Gata4* in mice with TAC

**Figure 4:**
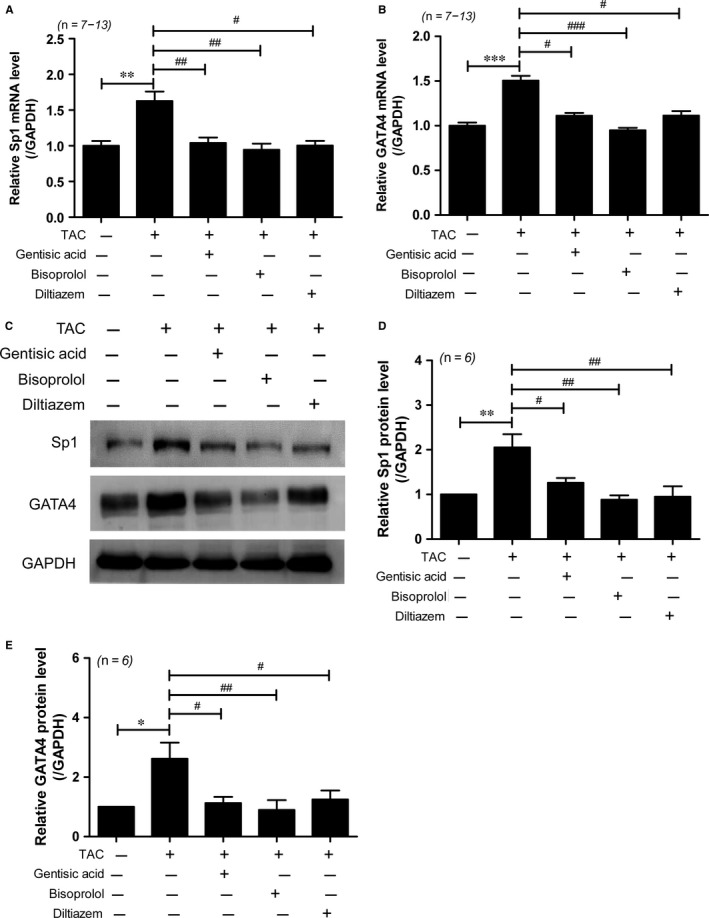
Gentisic acid reduces GATA4 and Sp1 expression in mice with transverse aortic constriction (TAC). Total RNA was isolated from the hearts of mice in the sham, TAC, TAC + gentisic acid, TAC + bisoprolol, and TAC + diltiazem groups. Levels of *Sp1* (A) and *Gata4* (B) transcripts were determined by RT‐qPCR and normalized to those of *Gapdh*. Data are means ± SE. C, Representative western blot images showing Sp1 and GATA4 protein levels in the hearts of mice. GAPDH was used as a loading control. The images were cropped from the same blot. D and E, Sp1 and GATA4 protein expression was quantified using densitometry. *P <* *0.05, **P <* *0.01, and ***P <* *0.001; ^#^ *P *<* *0.05, ^##^ *P *<* *0.01, and ^###^ *P *<* *0.001 vs the TAC group

We previously reported that gentisic acid is among the phenolic compounds present in *Dendropanax morbifera* branch extracts, and that such extracts attenuate isoproterenol‐induced Sp1 and GATA4 expression (paper under review). To determine whether gentisic acid alone affects these transcription factors, we examined their expression by RT‐qPCR and western blotting. *Sp1* and *Gata4* mRNA levels were significantly increased in response to TAC and suppressed by gentisic acid treatment (Figure 4A,B). Administration of bisoprolol or diltiazem also effectively reduced expression of these transcripts. Similar results were observed at the protein level for all three drugs. Western blot analysis revealed that Sp1 and GATA4 protein expression was induced by TAC, and treatment with gentisic acid, bisoprolol or diltiazem reduced this effect (Figure 4C‐E).

### 3.4 Gentisic acid suppresses the ERK1/2 MAPK signalling pathway in mice with TAC

**Figure 5:**
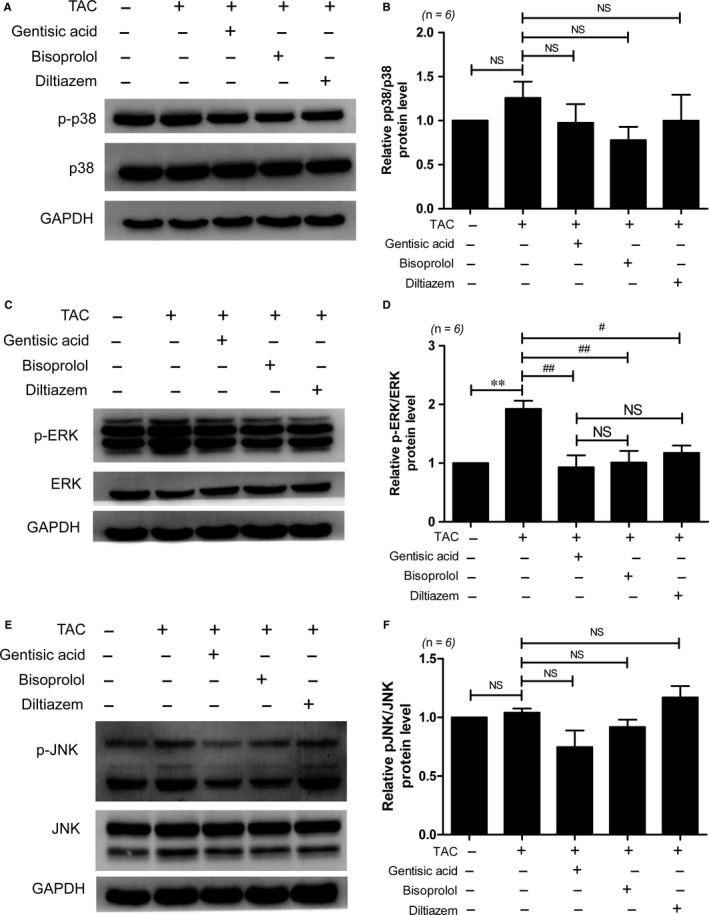
Gentisic acid attenuates ERK1/2 phosphorylation in mice with transverse aortic constriction (TAC). Representative immunoblots of phosphorylated (p‐)p38 MAPK (A), p‐ERK1/2 MAPK (C), and p‐JNK1/2 (E) levels in the sham, TAC, and drug‐treated TAC groups are shown. Levels of p‐p38 (B), p‐ERK1/2 (D), and p‐JNK1/2 (F) proteins were quantified using densitometry. Data are means ± SE. **P <* *0.01; ^#^ *P *<* *0.05 and ^##^ *P *<* *0.01 vs the TAC group; NS, not significant

To explore the regulatory mechanism by which gentisic acid inhibits cardiac hypertrophy, we examined the MAPK signalling pathway. Phosphorylation of p38 was not increased in hearts subjected to TAC compared with those in the sham group (Figure 5A,B). However, ERK1/2 phosphorylation was significantly increased in response to TAC, approximately 2‐fold compared to the control group (Figure 5C,D). Gentisic acid treatment significantly reduced the increased ERK phosphorylation, relative to the control group. Bisoprolol and diltiazem treatment also significantly reduced ERK phosphorylation to control levels in TAC. There was no significant difference in ERK phosphorylation between the three drug treatment groups (Figure 5D). JNK1/2 phosphorylation in the heart tissue was not increased by TAC (Figure 5E,F). There was no significant difference when comparing the TAC vehicle group to the gentisic acid, bisoprolol or diltiazem drug‐treated groups (Figure 5E,F).

### 3.5 Gentisic acid attenuates cardiac fibrosis in mice with TAC

**Figure 6:**
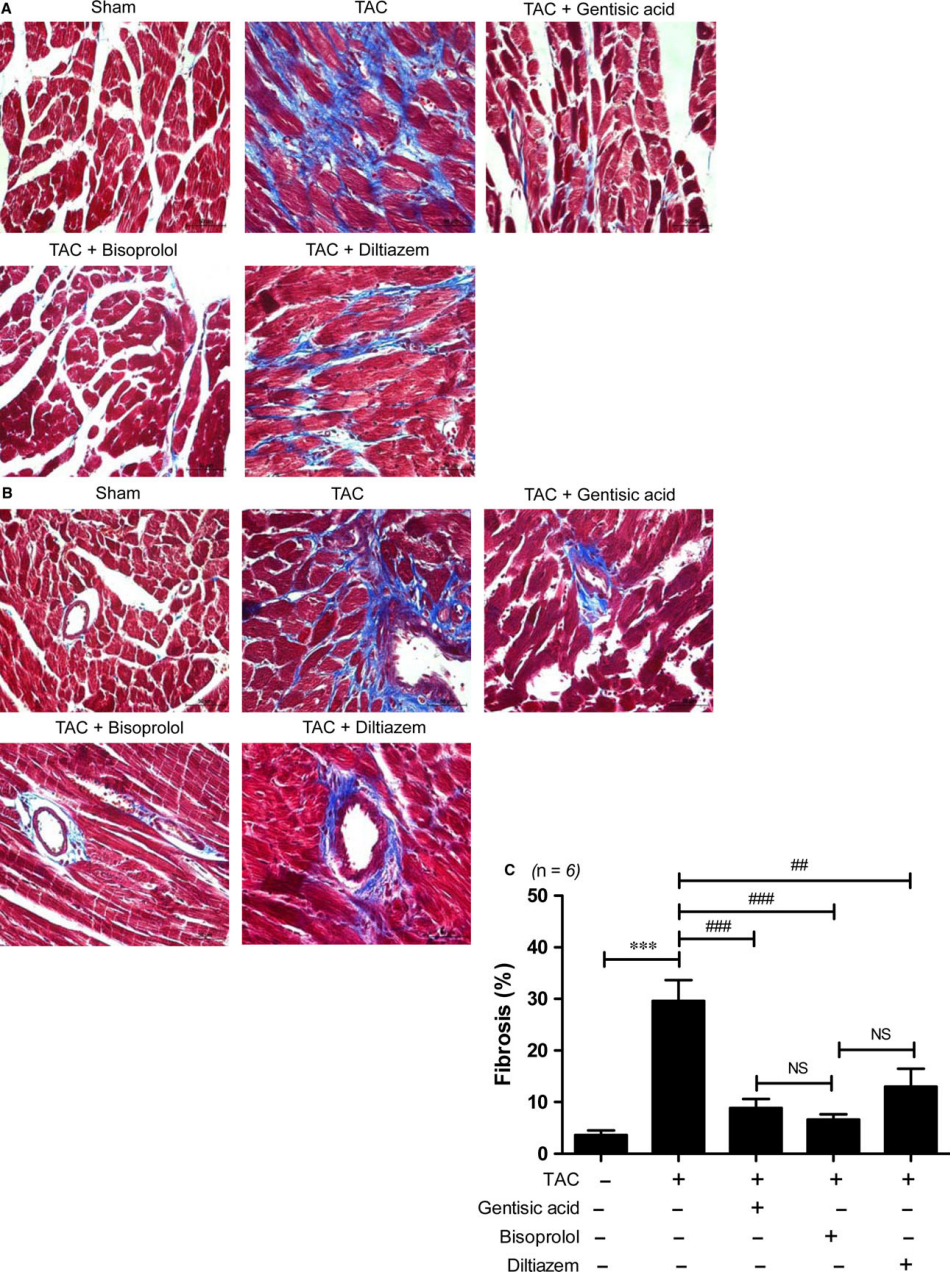
Gentisic acid reduces interstitial and perivascular fibrosis in mice with transverse aortic constriction (TAC). Representative images of Masson's trichrome staining of the hearts of mice in the sham and TAC + vehicle, gentisic acid, bisoprolol, or diltiazem groups. Scale bar = 50 μm. A, Interstitial fibrosis and B, perivascular fibrosis are shown. Fibrotic areas are stained blue. C, Fibrotic areas in specimens from the sham, TAC, and drug‐treated TAC groups were quantified (n = 6 per group). Data are means ± SE. ***P <* *0.001; ^##^ *P *<* *0.01 and ^###^ *P *<* *0.001 vs the TAC group

**Figure 7:**
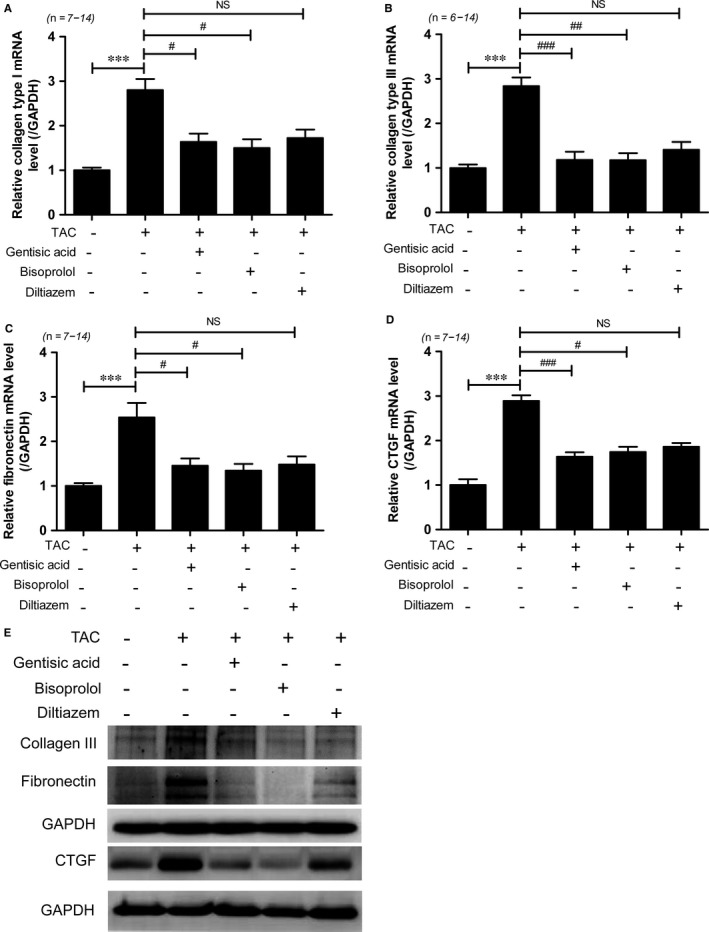
Gentisic acid attenuates expression of cardiac fibrosis marker genes in mice with TAC. Levels of collagen type I (A), collagen type III (B), fibronectin (C), and *Ctgf* (D) transcripts normalized to those of *Gapdh*. Data are means ± SE. ***P <* *0.001; ^#^ *P *<* *0.05, ^##^ *P *<* *0.01, and ^###^ *P *<* *0.001 vs the TAC group. E, Representative immunoblots for collagen III, fibronectin, and connective tissue growth factor (CTGF)

To determine whether gentisic acid decreases cardiac fibrosis following TAC, we performed Masson's trichrome staining. As shown in Figure 6A,B, a greater degree of cardiac interstitial and perivascular fibrosis was observed in the TAC group than the sham group. Compared to the vehicle control treatment, administration of gentisic acid, bisoprolol or diltiazem significantly reduced collagen accumulation in the hearts of mice with TAC. However, the extent of myocardial and perivascular fibrosis was greater in the diltiazem treatment group than the gentisic acid and bisoprolol groups. In particular, collagen accumulation around vessels was prominent in 4 of the 6 samples tested from the diltiazem‐treated TAC group. Although no significant difference in the suppression of cardiac fibrosis was evident among the drugs tested, Masson's trichrome staining suggested that bisoprolol was the most effective of the three in this respect. We next investigated the expression of fibrosis marker genes. Cardiac mRNA levels of collagen type I, collagen type III, fibronectin and *Ctgf* were higher in the TAC group than the sham group, and these increases were significantly reduced by gentisic acid treatment (Figure 7A‐D). The anti‐fibrotic effect of gentisic acid was similar to that of bisoprolol. Moreover, western blot analysis showed that treatment with gentisic acid, bisoprolol or diltiazem attenuated the TAC‐mediated upregulation of collagen III, fibronectin, and CTGF proteins in heart tissue (Figure 7E and Figure S2).

**Figure 8:**
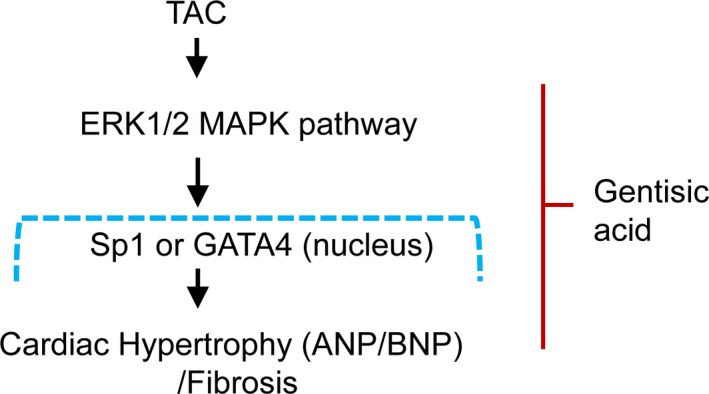
Gentisic acid attenuates cardiac hypertrophy and fibrosis through downregulation of the ERK1/2 pathway. Pressure overload, such as transverse aortic constriction (TAC), activates the ERK1/2 signalling pathway in the heart. Given TAC stimulation, in addition to the increase in GATA4 or Sp1 expression, atrial natriuretic peptide (ANP) and brain natriuretic peptide (BNP) expression are also elevated. In the cardiac hypertrophy mouse model, gentisic acid administration ameliorates cardiac hypertrophy and fibrosis through suppressed activation of ERK1/2 and downregulation of Sp1 or GATA4

## 4 DISCUSSION

In this study, we investigated the effects of gentisic acid treatment on a mouse model of pressure overload‐induced cardiac hypertrophy and fibrosis, and compared them to those of the calcium channel blocker diltiazem and beta blocker bisoprolol. We demonstrated that gentisic acid significantly inhibited TAC‐induced cardiac hypertrophy and fibrosis, with a therapeutic efficacy comparable to that of bisoprolol and diltiazem.

Recently, we reported that *Dendropanax morbifera* branch extract prevents cardiomyocyte hypertrophy. Among the 16 phenolic compounds identified in this extract, gentisic acid was found to be relatively abundant; however, its effect on pathological hypertrophy and fibrosis was not examined in an animal model. In the present study, approximately 5 weeks after the TAC procedure, marked cardiac hypertrophy and remodelling were evident, including increased gross heart mass, cross‐sectional area, and thickness of the left ventricular septum and posterior wall. Gentisic acid (100 mg/kg/d) treatment of mice with TAC restored cardiac wall thickness to the level observed in the sham group, as determined by echocardiographic parameters. The anti‐hypertrophic effect of gentisic acid was similar to that of bisoprolol (5 mg/kg/d) and diltiazem (10 mg/kg/d). Thus, gentisic acid represents a promising candidate therapy for the reduction of cardiac hypertrophy. These data are consistent with the findings of Xiang et al,^26^ who reported that administration of various doses of bisoprolol (2.5, 5, or 10 mg/kg/d) for 8 weeks diminishes TAC‐induced cardiac hypertrophy. The dose of diltiazem used in the present study is comparable to that used (360 mg/d, i.e., 5 mg/kg/d) in an investigation of the therapeutic effect of preclinical administration of this drug to individuals at risk of hypertrophic cardiomyopathy.^28^ We selected diltiazem as a reference drug due to its demonstrated ability to prevent hypertrophic cardiomyopathy in mice.^29^ In addition to diltiazem, there have been several reports showing that other calcium channel blockers, such as benidipine and amlodipine, ameliorate cardiac remodelling and myocardial hypertrophy.^30^, ^31^

Gentisic acid treatment also attenuated the increased cardiac expression of hypertrophic marker genes, including *Anp*,* Bnp*, and skeletal α‐actin, induced by TAC. In our previous study, *Dendropanax morbifera* branch extract was found to ameliorate isoproterenol‐induced Sp1 and GATA4 expression in cardiomyoblasts. In the current investigation, we decided to determine whether gentisic acid from this extract affects the Sp1/GATA4 pathway in pressure overload‐induced cardiac hypertrophy. Expression of these transcription factors was significantly increased in the TAC group compared to the sham group. Thus, we have shown that Sp1 and GATA4 are upregulated in response to hypertrophic stimuli both in vitro and in vivo. These findings are consistent with a previous study^32^ and similar to those of Azakie et al,^33^ who reported that GATA4 and Sp1 protein levels are increased in a neonatal lamb cardiac shunt model of biventricular hypertrophy. These data suggest that the transcription factor activity of GATA4 and Sp1 exacerbates cardiac hypertrophy. However, treatment of mice having undergone TAC surgery with gentisic acid, bisoprolol, or diltiazem suppressed such increased Sp1 and GATA4 expression. It is very well known that GATA4, whose downstream targets include *Anp* and *Bnp*, plays an important regulatory role in the induction of cardiac hypertrophy.^34^ We therefore suggest that gentisic acid is able to suppress cardiac hypertrophy through the Sp1/GATA4/ANP axis.

MAPKs are known to have important functions in heart development, cardiac hypertrophy, and pathological cardiac remodelling.^7^, ^35^ Our study demonstrated that gentisic acid treatment inhibited the TAC‐induced phosphorylation of ERK1/2 in cardiac tissue. However, p38 and JNK1/2 activation was not clearly observed in hearts following 5 weeks of TAC. This activation of ERK1/2 is consistent with reports showing that TAC triggers ERK1/2 phosphorylation.^36^, ^37^, ^38^ The inhibitory effect of gentisic acid on ERK1/2 activation was almost the same as that noted following administration of bisoprolol or diltiazem. This implies that gentisic acid can be used to reduce pathological cardiac hypertrophy and is not inferior to a clinically available beta‐blocker and calcium channel blocker in this regard.

Interestingly, in the present study, gentisic acid administration significantly attenuated cardiac fibrosis in mice with TAC compared with the sham surgery control group. Interstitial and perivascular fibrosis were evident in heart tissue after 5 weeks of TAC. Masson's trichrome staining revealed that, of the three drugs tested, levels of fibrosis were lowest following bisoprolol treatment and highest after diltiazem administration. This result is consistent with the demonstration by Xiang et al^26^ that bisoprolol at a dose of 5 mg/kg/d prevents TAC‐induced cardiac fibrosis. However, considering the dosage of bisoprolol used in clinical practice (5 mg/d for adults),^39^ that employed in our animal experiments was very high (70 times greater), possibly explaining the stronger reduction in TAC‐associated cardiac fibrosis noted using this drug. In contrast, the diltiazem dose used in our investigation was three times higher than that given to patients (90 mg twice daily).^40^

Gentisic acid treatment also reduced cardiac expression of collagen type I, collagen type III, fibronectin, and CTGF in mice with TAC, and bisoprolol and diltiazem administration had similar antifibrotic effects. Gentisic acid is a metabolite of aspirin (salicylic acid) and acts as an antioxidant.^16^ It has the potential to ameliorate conditions related to cardiovascular diseases, such as hypertension, atherosclerosis and dyslipidaemia.^20^ For example, it has been reported to diminish the glucose autoxidation‐associated oxidative modification of low‐density lipoprotein, leading to reduced atherogenesis.^41^ Moreover, a recent study has reported that gentisic acid is superior to norepinephrine in preventing the development of lactic acidemia in a canine model of septic shock.^42^ Our findings that gentisic acid exerts antihypertrophic and antifibrotic effects demonstrate its potential to prevent or treat pathological cardiovascular diseases.

### 4.1 Limitations

One limitation of this study is that only a high dose of gentisic acid was tested (100 mg/kg/d). It will be necessary to establish whether a lower concentration has similar effects on cardiac hypertrophy and fibrosis. Gentisic acid is relatively rapidly excreted in urine after oral ingestion. Previous work using dogs has shown that most of a single oral dose of 190 mg/kg is excreted on the day of administration.^43^ This implies that in the present study, the dose used was unlikely to have resulted in the accumulation of gentisic acid and any associated toxicity, in the mice tested. Therefore, toxicity testing is essential in future when gentisic acid can be used in drug development.^44^ Indeed, we have confirmed that oral administration of gentisic acid (2000 mg/kg) to female mice did not show any toxicity in the heart, liver, and kidney as determined by H&E staining (Figure S3). Our acute toxicity test showed an LC_50_ of greater than 2000 mg/kg for gentisic acid with no appearance of inflammatory or histological abnormalities within 2 weeks of the study. In addition, we need to repeat the dose toxicity testing to ensure safety for human use. Considering that calcium channel blockers are not recommended because they do not improve heart function in patients with heart failure with reduced ejection fraction,^45^ it suggests that gentisic acid may be replaced by a treatment for acute heart failure patients.

In conclusion, gentisic acid effectively inhibited cardiac hypertrophy and fibrosis in a mouse model of pressure overload through downregulation of Sp1/GATA4 expression and ERK1/2 signalling pathways. Furthermore, the beneficial effect of gentisic acid was comparable to that of bisoprolol. Our results suggest that gentisic acid may be used for the treatment of cardiac hypertrophy and fibrosis.

## CONFLICTS OF INTEREST

None of the authors have any conflicts of interest to declare.

## AUTHOR CONTRIBUTIONS

S.S. and H.J.K contributed to the conception and design of the experiments; S.S., L.J., Y.R., S.Y.C., and G.R.K. performed the experiments; S.S., Y.R., H.J.K., and M.H.J. performed data analysis and interpretation; S.S. and H.J.K. wrote and revised the manuscript.
