# Supplementary material for: Gentisic acid attenuates pressure overload‐induced cardiac hypertrophy and fibrosis in mice through inhibition of the ERK1/2 pathway
Source: J Cell Mol Med. 2018 Sep 6;22(12):5964–77. doi: 10.1111/jcmm.13869 (PMC6237595; doi:10.1111/jcmm.13869)

# Gentisic acid attenuates pressure overload-induced cardiac hypertrophy and fibrosis in mice through inhibition of the ERK1/2 pathway

Simei Sun[1,2,3], Hae Jin Kee[1,2*], Li Jin[4], Yuhee Ryu[1,2], Sin Young Choi[1,2], Gwi Ran Kim[1,2] and Myung Ho Jeong[1,2*]

[1]Heart Research Center of Chonnam National University Hospital, Gwangju, 61469, Republic of Korea

[2]Hypertension Heart Failure Research Center, Chonnam National University Hospital, Gwangju, 61469, Republic of Korea

[3]Molecular Medicine, Brain Korea 21 PLUS, Chonnam National University Graduate School, Gwangju, 61469, Republic of Korea

[4]The Second Affiliated Hospital & Yuying Children's Hospital Wenzhou Medical University. Wenzhou, 325027, China

## Supplementary Method

### Acute toxicity study

The acute toxicity experiment was performed using female mice weighing approximately 25 g. Mice were grouped into two groups: control (vehicle, n = 6) and gentisic acid (2000 mg/kg, n = 6) treatment. A single-administration of gentisic acid was given to the mice. Subsequently, the mice were observed for survival and behavioral changes for 14 days. Mice were sacrificed, and paraffin blocks were made to analyze the heart, liver, and kidney tissues. Pathologists analyzed whether inflammation and adverse events occurred after H&E staining.

## Supplementary Figure Legends

**Supplementary Figure 1. Echocardiographic parameters of mice with TAC**

Left ventricular hypertrophy in mice was determined by echocardiography after 3 weeks of drug administration beginning 2 weeks after TAC surgery. (**A**) Left ventricular internal diameter end systole (LVESD); (**B**) left ventricular internal diameter end diastole (LVEDD).

**Supplementary Figure 2. Gentisic acid attenuates expression of proteins encoded by cardiac fibrosis marker genes in mice with TAC**

Levels of collagen type III (**A**), fibronectin (**B**), and CTGF (**C**) proteins were evaluated by western blot analysis. Data are means ± SE. $^{*}P < 0.05$, $^{**}P < 0.01$, and $^{***}P < 0.001$; $^{\#}P < 0.05$, $^{\#\#}P < 0.01$, and $^{\#\#\#}P < 0.001$ versus the TAC group.

**Supplementary Figure 3. Gentisic acid does not affect acute toxicity in the hearts, livers, and kidneys of mice**

Female mice were orally administrated with vehicle or gentisic acid (2000 mg/kg). After two weeks, mice were sacrificed, and a histological study was performed using hematoxylin and eosin (H&E) staining. Representative images of H&E staining in the hearts (**A**), livers (**B**), and kidneys (**C**) of vehicle and gentisic acid-treated mice. Scale bar, 100 μm.

# Supplementary Figure 1

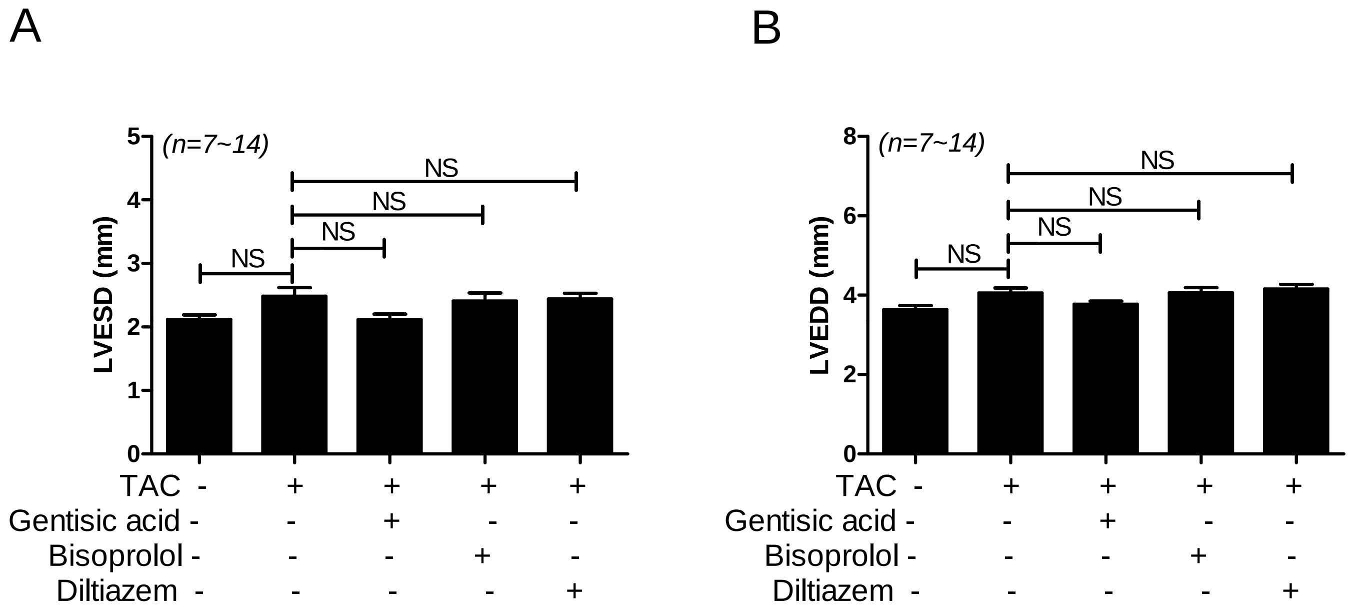

# Supplementary Figure 2

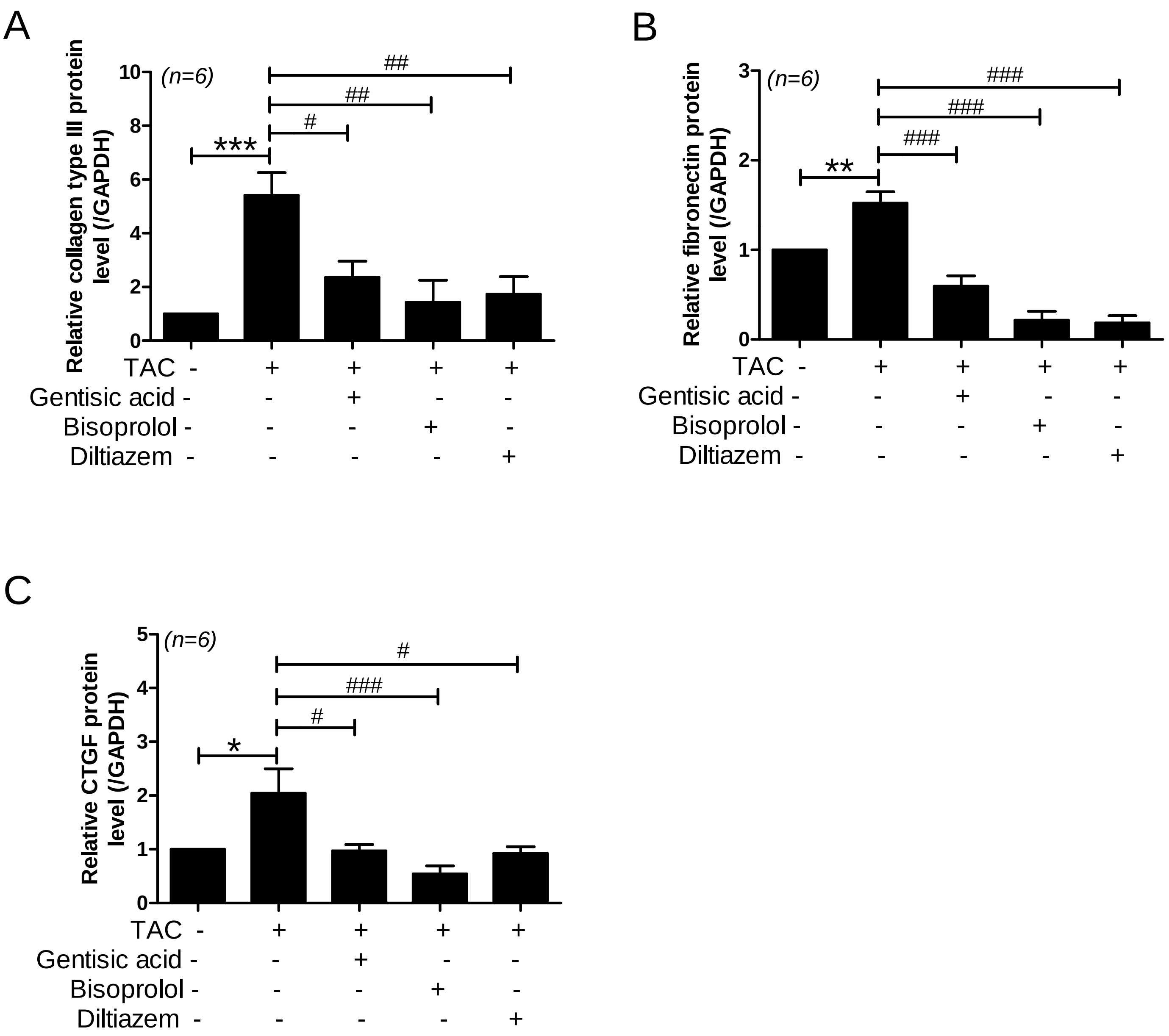

Supplementary Figure 3

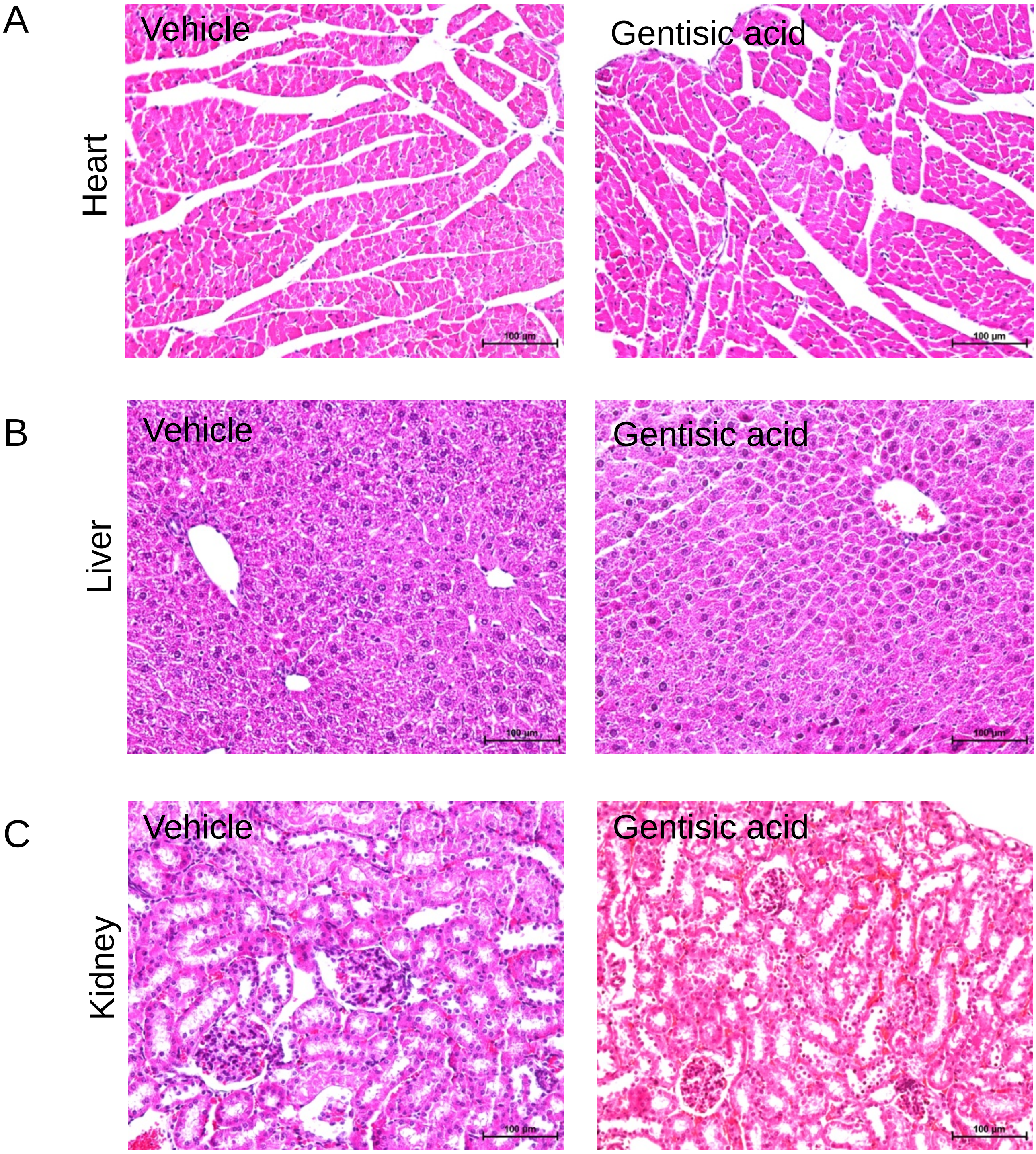

Supplement: Supplementary file 1 [file JCMM-22-5964-s001.pdf]
